# Formulation of a Phenol-Rich Extract from Unripe Olives (*Olea europaea* L.) in Microemulsion to Improve Its Solubility and Intestinal Permeability

**DOI:** 10.3390/molecules25143198

**Published:** 2020-07-13

**Authors:** Lorenzo Cecchi, Vieri Piazzini, Mario D’Ambrosio, Cristina Luceri, Federica Rocco, Marzia Innocenti, Giulia Vanti, Nadia Mulinacci, Maria Camilla Bergonzi

**Affiliations:** 1 Department of Neuroscience, Psychology, Drug Research and Child Health (NEUROFARBA), Section of Pharmaceutical and Nutraceutical Sciences, University of Florence, via U. Schiff 6, Sesto Fiorentino, 50019 Florence, Italy; lo.cecchi@unifi.it (L.C.); fede.rocco12@gmail.com (F.R.); marzia.innocenti@unifi.it (M.I.); nadia.mulinacci@unifi.it (N.M.); 2 Department of Chemistry, University of Florence, via U. Schiff 6, Sesto Fiorentino, 50019 Florence, Italy; vieri.piazzini@unifi.it (V.P.); giulia.vanti@unifi.it (G.V.); 3 Department of Neurosciences, Psychology, Drug Research and Child Health (NEUROFARBA), Section of Pharmacology and Toxicology, University of Florence, Viale Pieraccini 6, 50139 Florence, Italy; mario.dambrosio@unifi.it (M.D.); cristina.luceri@unifi.it (C.L.)

**Keywords:** *Olea europaea* L., phenolic compounds, microemulsion, PAMPA, caco-2, oleuropein, hydroxytyrosol, permeability, ligstroside, verbascoside

## Abstract

The beneficial properties of phenolic compounds from *Olea europaea* L. are well-known. An olive extract (OE) was prepared from unripe olives (Moraiolo cultivar). The study aimed to formulate OE into a microemulsion (ME) in oral dosage form. OE was extracted from olives with EtOH:H_2_O (80:20) and characterized by HPLC-DAD. ME composition was stated by a solubility and pseudo-ternary diagram. The ME was chemically and physically characterized, and its stability at 4 °C was analyzed for three months. The ability of the formulation to ameliorate the solubility and the intestinal permeability of OE was evaluated by a Parallel Artificial Membrane Permeability Assay (PAMPA) assay and Caco-2 cells. The total phenolic content of the extract was 39% *w*/*w*. The main constituent was oleuropein (31.0%), together with ligstroside (3.1%) and verbascoside (2.4%). The ME was prepared using Capryol 90 as the oily phase, and Cremophor EL and Transcutol (2:1) as surfactant and co-surfactant, respectively. ME droplet size was 14.03 ± 1.36 nm, PdI 0.20 ± 0.08, ζ-potential −1.16 ± 0.48. Stability of ME was confirmed for at least three months. The formulation was loaded with 35 mg/mL of OE, increasing the solubility of the extract by about four times. The enhanced permeability of OE was evaluated by PAMPA, as demonstrated by the Pe value (1.44 ± 0.83 × 10^−6^ cm/s for OE hydroalcoholic solution, 3.74 ± 0.34 × 10^−6^ cm/s for OE-ME). Caco-2 cell transport studies confirmed the same results: P_app_ was 16.14 ± 0.05 × 10^−6^ cm/s for OE solution and 26.99 ± 0.45 × 10^−6^ cm/s for OE-ME. ME proved to be a suitable formulation for oral delivery.

## 1. Introduction

The beneficial health properties associated to a regular consumption of extra virgin olive oil (EVOO) and other food products derived from the olive tree (*Olea europaea* L.) are well-known, mainly thanks to the presence of specific phenolic compounds. For these reasons, natural remedies against diseases, nutraceutical ingredients, and food supplements based on phenol-rich extracts from different parts of the olive tree have been proposed for the protection of people’s well-being [1,2,3,4].

The main phenolic compound present in the olive fruits is the secoiridoid oleuropein, followed by other secoiridoids, namely ligstroside, nuzhenide (only present in seeds), and demethyloleuropein (which appears in late ripening stages), and by the phenylpropanoid derivative verbascoside [5,6,7,8]. Their concentration is affected by ripening stage and the part of the fruit [5,9,10,11]. Oleuropein reaches the maximum concentration (up to 14% *w*/*w* dm) in young fruits [10]. Then, during the green maturation, its level, as well as those of verbascoside and ligstroside, constantly decreases [5,12,13,14]. 

Several beneficial properties have been described for the main phenolic compounds present in unripe olive. Oleuropein showed potential antioxidant, anti-inflammatory, anti-atherogenic, anti-cancer, anti-viral, anti-aging, and neuroprotective properties [10,15,16,17]. Verbascoside, also known as acteoside or kusaginin [18], is a bio-active molecule well-known for its antioxidant, anti-inflammatory, antibacterial, antifungal, potential anticancer, and anti-tuberculosis properties [18,19,20,21,22,23]. In a recent study, ligstroside showed beneficial effects against Alzheimer’s disease and the capability in expanding the lifespan and enhancing the cognitive function of aged mice in vivo [24].

The phytocomplex, that is a whole or partially purified extract of a plant constituent, offers greater efficacy and more advantages than a single isolated ingredient, thanks to synergistic interactions [25,26], and this is also true for the biological effects exerted by phenolic compounds from *Olea europaea* L. [26]. All this evidence makes dried unripe olives, very rich in verbascoside, ligstroside and, particularly, oleuropein (up to 10% *w*/*w*) and total phenolic content (up to 18% *w*/*w*), a very interesting raw material for preparing a new ingredient for food supplement very rich in phenols from *Olea europaea* L. [27]. This is especially true if olives are harvested at a very early stage, before any stone lignification, with this fact making the fruit easy to mince in order to obtain the ingredient.

Oleuropein and ligstroside are absorbed and subjected to phase-II metabolism in humans and then they are extensively metabolized to yield many other metabolites whose specific role in the biological properties attributed to oleuropein and ligstroside are yet to be clarified [16]. A study on rats supplemented by a phenolic extract of olive cake, suggests that these molecules and their metabolites are widely distributed in multiple organs or further metabolized [28]. The high correlation found in plasma among the metabolites of hydroxytyrosol (glucuronide and sulfate) and of oleuropein aglycon glucuronide was suggested as indicative of a variable efficiency of gastric hydrolysis in each individual [16]. The exposure to hydroxytyrosol resulted in a significant degree from ingested oleuropein aglycone contained in EVOO [17]. In terms of gender research, it has been pointed out that hydroxytyrosol metabolism is more efficient in female than in males [29].

Novel nanoformulations based on drug delivery systems are designed with the aim of enhancing bioavailability, solubility, physicochemical stability, intestinal permeation, resident time in the gastrointestinal tract, and controlled release of natural products [30,31,32].

In a recent study, zwitterionic liposomes containing phenols from *Olea europaea* L. were characterized for increasing phenols’ bioavailability, but standards and not extracts were used [33]. Other studies, dealing with preparation of liposomes [34], W/O emulsion, microemulsion [35], and mPEG-PLGA nanoparticles [36], only focused their attention on hydroxytyrosol, while other ones focused their attention on extracts from olive leaves [37]. However, to the author’s knowledge, no studies have investigated on the possibility to incorporate the phytocomplex of unripe olives in a drug-delivery system.

Amongst the various drug delivery systems, the microemulsion (ME) is considered an ideal alternative for the oral delivery of olive extract. ME is able to transform the dried extract into an oral dosage form to overcome the limited oral bioavailability, thereby increasing the therapeutic efficacy of the extract. Oil in water microemulsions have been formulated with food acceptable components to increase the solubility, stability, and ameliorate the intestinal permeability of the extract’s constituents [38,39,40]. Additionally, several excipients used in these systems, including Cremophor, Tween 80, Labrasol, and Transcutol, could inhibit the function of P-gp [41,42,43]. Usually, the permeability of a compound or of a series of compounds present in an extract or formulation is assessed using cell cultures, and the Caco-2 monolayers models are the most widely used so far [44,45,46]. However, the long cell growth cycle and the high costs limit its use as a high throughput tool. For this reason, other models aimed to simulate gastrointestinal permeability, maintaining a good correlation with the in vivo permeability but with lower costs, have been developed using artificial membranes. The most used of this type of in vitro assay is the Parallel Artificial Membrane Permeability Assay (PAMPA) [45,47,48,49]. Recently, the PAMPA model has also been successfully used and validated for testing the permeability of bioactive compounds from extracts of several species and their formulations (e.g., *Vitex agnus-castus*, *Silybum marianum*, *Chenopodium quinoa*, *Trigonella foenum-graecum*, and *Posidonia oceanica*) [45,49,50,51].

This study aimed to prepare a suitable and cheap drug-delivery-system to improve the bioavailability of phenolic compounds present in extracts from Tuscan unripe olives (cv Moraiolo) harvested before any stone lignification. A phenol-rich extract (OE) was prepared from freeze-dried olives using a hydroalcoholic solvent, and was characterized by HPLC-DAD. The more suitable ingredients of ME were selected after the solubility study in different vehicles. ME was chemically and physically characterized with DLS and HPLC-DAD analyses to evaluate the size, homogeneity, morphology, and encapsulation efficiency. Its stability was tested over three months and the permeation and transport studies were performed to determine the suitability of the formulation for oral delivery. Different in vitro models were applied, such PAMPA [47,51] and Caco-2 cell transport experiments.

## 2. Results and Discussion

### 2.1. Phenolic Composition and Solubility in Different Vehicles of the Phenolic Extract

**Figure 1:**
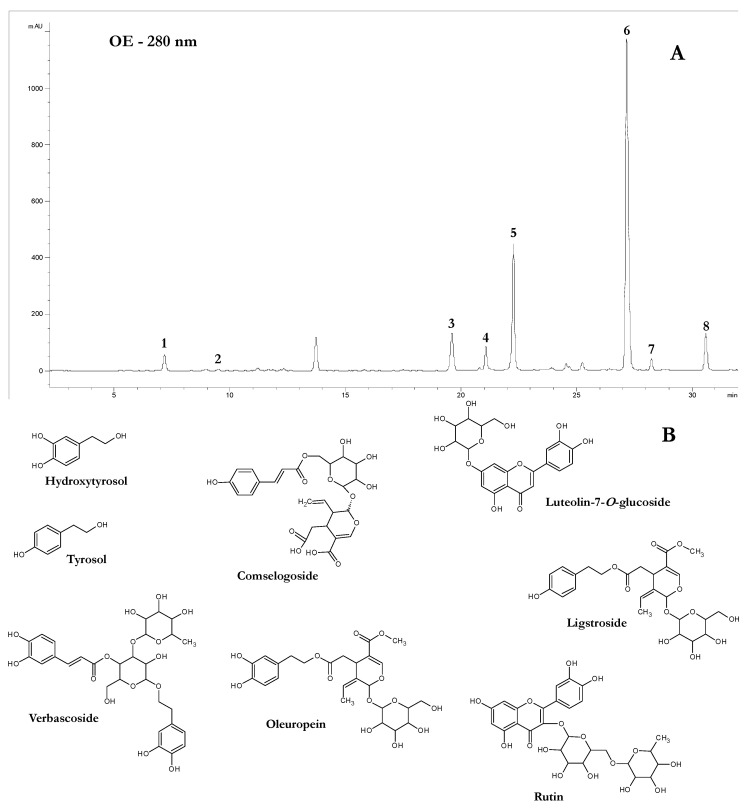
(**A**) Phenolic profile at 280 nm of the olive extract (OE). 1, hydroxytyrosol; 2, tyrosol; 3, rutin; 4, luteolin-7-O-glucoside; 5, verbascoside; 6, oleuropein; 7, comselogoside; 8, ligstroside. (**B**) The main phenolic compounds detected in the olive extract.

The phenolic composition of the olive extract was characterized using a chromatographic approach already applied in our studies for analyzing the phenolic fraction of olives and derivatives [52,53], but avoiding the use of the internal standard. Consequently, the quantitative analysis was carried out using external calibration curves, instead of the internal standard method applied in the above cited papers that required the addition of syringic acid (ISTD) during the preparation of the OE. The presence of a molecule other than those naturally present in the extract would have interfered with and modified the final results of the study. The phenolic composition of the OE is reported in Table 1, while Figure 1 shows the phenolic profile of the OE (Figure 1A) and the chemical structure of the quantified phenolic compounds (Figure 1B).

The concentration of total phenols in the OE is very high (approx. 39% *w*/*w*). In particular, the highest concentrations were for oleuropein (31.0% *w*/*w* of the weight of the extract, representing approx. 80% of the total phenolic content), verbascoside (2.3% *w*/*w*, approx. 6% of the total phenolic content), and ligstroside (3.1% *w*/*w*, approx. 8% of the total phenolic content). To the author’s knowledge, the concentration of phenolic compounds in OE is higher than in other commercial extracts from *Olea europaea* L. As far as the widely used olive leaves extracts, some papers reported variable phenolic contents, usually lower than the values reached with the extraction of unripe dried olives selected for this study: in particular, oleuropein content was found in very low amounts, e.g., 0.077% *w*/*w* in a paper [54], and ranging from 2.1% to 20.4% in other papers [4,55,56,57,58,59,60], while the total phenolic content ranging 3.4% to 31.6% [4,56,57,58,59,60], and reaching 38.4% only in one paper [55]. The typical phytocomplex of olive leaves is different from that of the OE, which shows unique characteristics from both qualitative and quantitative points of view. The yields of the extract on the unripe olives weight (40%, see Section 3.2) and the concentration of phenols per gram of extract are very high, and thus OE represents a new and interesting nutraceutical ingredient that could be used for the protection of people’s well-being and to allow producers the possibility of diversifying their production, and thus new possibilities of income, in a period of the year when olive oil production has not yet commenced. Interestingly, the required amount of OE for the preparation of a microemulsion (see next paragraphs) can be obtained without negatively affecting the olive oil yields in a significantly way.

After the chemical characterization, solubility studies of olive extract were performed in water and in different surfactants and lipophilic solvents to select appropriate constituents for ME formulation. Table 2 shows that the vehicles differently affect the solubility of phenolic compounds, if compared with water: Captex 300, Captex 355, Labrafac, Labrafilm 1944, Labrafilm 2125, and Lauroglycol 90 showed a poor solubilization capability, while Transcutol, Labrasol ALF, and Cremophor EL showed the best solubilization capability, higher than water. Transcutol solubilizes a quantity of phenolic compounds that is approximately triple compared to water. These data suggest the possibility to strongly improve the solubility of OE phenolic compounds in comparison with pure water by using an optimized combination of the above vehicles in an ME.

### 2.2. Pseudo-Ternary Phase Diagram

**Figure 2:**
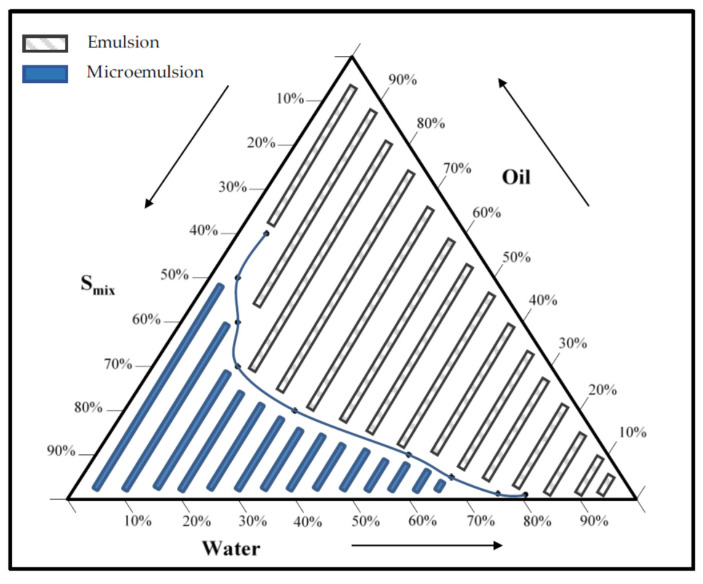
Pseudo-ternary phase diagram. The dark area represents the ME existence range and the grey area means crude emulsion range.

Based on the above results, Capryol 90 was selected as oil phase, and Cremophor EL and Transcutol as surfactant and co-surfactant, respectively. All the selected components have already been used and approved for oral administration purposes. Capryol 90 is propylene glycol monocaprylate and was already selected as oily phase in other studies due to its relative easiness of emulsification [61]. Capryol 90 has been investigated extensively as the oil phase for the development and optimization of nanoemulsions/microemulsions/Self-Nanoemulsifying Drug Delivery Systems (SNEDDS)/Self-Microemulsifying Drug Delivery Systems (SMEDDS) of various poorly soluble drugs both in vitro and in vivo [62]. Cremophor EL is a complex mixture of hydrophobic and hydrophilic components, with the main components being glycerol polyethylene glycol ricinoleate and glycerol ethoxylates, respectively. It had a very good ability to emulsify Capryol 90, as previously reported [61]. Transcutol is a hydrophilic co-surfactant and increases the spontaneity of the ME formation. It improves the emulsification of surfactants by penetrating the interfacial surfactant monolayer effectively, and it has a superior solubilizing potential performance. Cremophor EL and Transcutol were mixed at different ratios under vigorous stirring to obtain the surfactant mixture (S_mix_). Then, the pseudo-ternary phase diagram was constructed by the water titration technique, using different combinations of Capryol 90 and each S_mix_. Figure 2 shows the obtained pseudo-ternary phase diagram.

The final microemulsion was constituted by 1% Capryol 90 (oil), 12.7% Cremophor EL (surfactant), and 6.3% Transcutol (co-surfactant), in addition to water (80%).

### 2.3. Solubility of Olive Extract into Microemulsion

After the elaboration of a pseudo-ternary phase diagram, the maximum loading content of OE into the formulation was evaluated by adding an increasing amount of extract to the ME under stirring. The formulation was able to incorporate up to 35 mg/mL of OE, corresponding to 13.71 ± 0.01 mg/mL of the identified phenolic molecules, without phase separation or precipitation of the extract. The OE is a hydro-alcoholic extract, partially soluble in water. It contains different phenolic compounds, mainly oleuropein, verbascoside, and ligstroside, with different structure and aqueous solubility. The ME increases the solubility of OE more than three times due to the presence of oil phase and tensides, which can also realize a micellar solubilization of the lipophilic compounds.

### 2.4. Particle Size, ζ-Potential Measurements and In Vitro Release Study

Empty and extract-loaded formulations were physically characterized by dynamic light scattering (DLS) and electrophoretic light scattering (ELS). The analyses confirmed the presence of a homogeneous system with narrow size distribution, low values of polydispersity index (PdI), and mean diameter (Table 3). The presence of the OE did not affect the physical properties of the system. ME showed very small particle size (<100 nm) which could promote the absorption by enterocytes and help to avoid the uptake by the cells of the reticuloendothelial system (RES) [63].

Then, developed ME represents a successful tool to incorporate OE and to significantly ameliorate its solubility, in that it is able to solubilize 35 mg/mL of OE without destabilization of the system.

**Figure 3:**
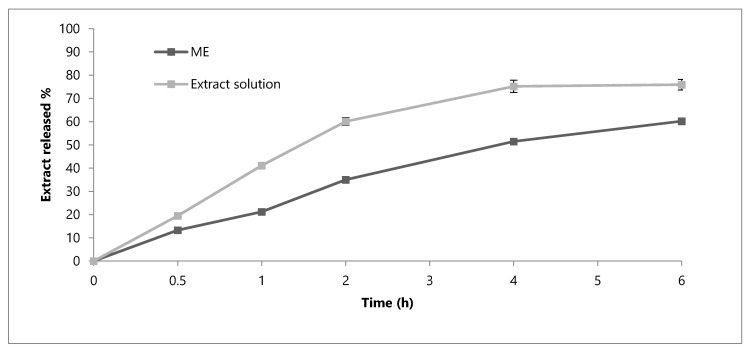
In vitro release profile of phenolic compounds from the OE-ME and OE ethanolic solution in Phosphate Buffered Saline (PBS) medium at pH 7.4. Each value is the mean ± SD of three separate determinations.

**Figure 4:**
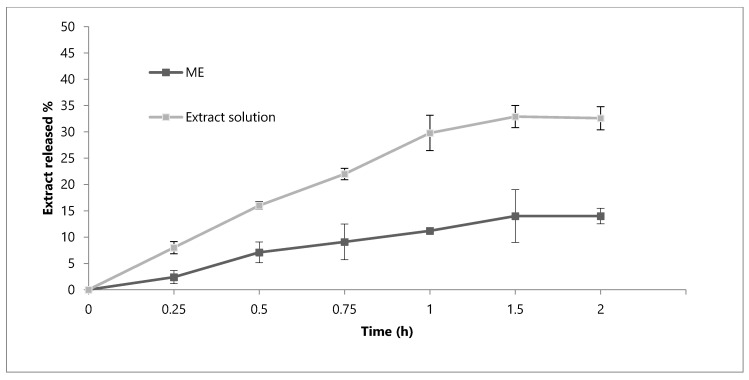
In vitro release profile of phenolic compounds from the OE-ME and OE ethanolic solution in Simulated Gastric Fluid (SGF) medium at pH 1.2. Each value is the mean ± SD of three separate determinations.

To further evaluate the appropriateness of the developed formulation for oral use, it is necessary to take into consideration the characteristics of the different environments that it can meet and the possible modifications of the loaded phenolic compounds. ME and hydroalcoholic solution (EtOH-H_2_O 70:30), both containing 35 mg/mL of extract, were compared and their behaviour has been taken into consideration to evaluate if the formulation could guarantee a prolonged release of the phenolic compounds. A first in vitro release study was carried out at the physiological pH (7.4), thus simulating the conditions that the formulation meets after its absorption (Figure 3). After 6 h, the percentage of phenolic compounds released from ME (60.2%) was lower than that obtained with the solution (75.9%). A similar behaviour was obtained in simulated gastric fluid (SGF, Figure 4). By comparing the ME and solution, the release of the extract is more gradual and prolonged in the case of ME. It can be noted that, after 2 h, the release of the extract by the solution corresponds to 32.6% of the phenolic compounds, while the formulation releases only the 14.0%.

**Figure 5:**
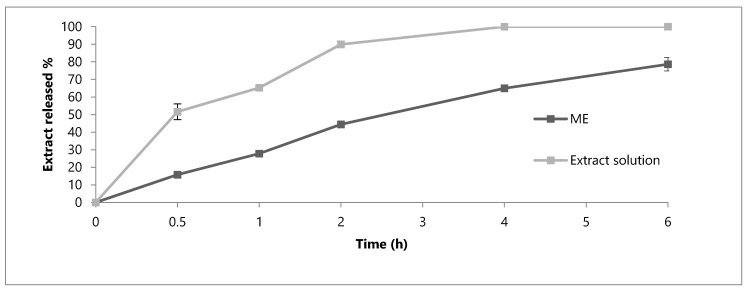
In vitro release profile of phenolic compounds from the OE-ME and OE ethanolic solution in Simulated Intestinal Fluid (SIF) medium at pH 6.8. Each value is the mean ± SD of three separate determinations.

The last in vitro release study was carried out in the SIF at pH 6.8 (Figure 5) in order to simulate the intestinal transit. In a comparison between the two formulations, ME showed a more prolonged release than solution. In this case, the quantities of released phenolic compounds are greater than in the other two cases: after 6 h, the percentage reaches 100% with the solution and 78.6% with the ME. Notably, in this condition, the release from the solution was 100% after 4 h, while the release from the ME is still increasing after 6 h, pointing to the capability of the ME to make the release much more gradual and prolonged than the solution.

ME achieves a gradual release and protects the OE from degradation in the acid environment, because it releases a smaller quantity of phenolic compounds compared to the solution and compared to the amount released in the other two media, i.e., PBS and SIF.

### 2.5. Chemical and Physical Stability During Storage

In order to estimate the stability of the formulation, the OE-ME was stored in sealed glass containers at 4 °C for three months. Periodically, chemical and physical stability were checked by monitoring transparency, phase separation, colour variation, as well as the changes in particle size, homogeneity, ζ-potential, and extract concentration by DLS and HPLC-DAD analyses.

The formulation proved to be stable during the whole duration of the test: no phase separation or creaming were observed. Moreover, size, homogeneity and ζ-potential value resulted unchanged after 3 months: size 12.94 ± 0.10 nm, PdI 0.13 ± 0.01, −1.10 ± 0.01 mV. The concentration of phenolic compounds was 13.01 ± 0.32 mg/mL, corresponding to 95% of the starting amount. Moreover, the composition of the phenolic compounds was unchanged, with oleuropein ca. 80%, ligstroside ca. 8%, and verbascoside ca. 6%. These findings evidenced the stability during the storage period of the developed formulation and the ability of ME to prevent the degradation of loaded compounds.

### 2.6. In Vitro Parallel Artificial Membrane Permeability Assay (PAMPA)

PAMPA represents a potential approach for the rapid assessment of passive transport permeability. PAMPA is performed to estimate passive transcellular permeability. It is a non-cell-based permeability model, but is considered robust, reproducible, and a helpful complement to the cellular permeability model for its speed, low cost, and versatility. A combination of the PAMPA and Caco-2 permeability model can synergistically provide invaluable permeability/absorption assessment of a drug. The assay could be applied not only in the pre-formulation studies of single molecules, but also to obtain information on the behavior of the extracts, and recently the authors applied the test to the study of compounds loaded into formulations, i.e., SLN, NLC, nanomicelles, and microemulsions [15,45,64,65].

The test was carried out in a 96-well, MultiScreen-IP PAMPA (Millipore corporation) filter plate. The ability of compounds to diffuse from a donor compartment into an acceptor compartment was evaluated. The P_e_ of extract solution (EtOH:PBS 70:30) was 1.44 ± 0.83 × 10^−6^ cm/s while the P_e_ of OE-ME was 3.74 ± 0.34 × 10^−6^ cm/s, *p* = 0.0286. The formulation improved the passive permeation of extract across the simulated membrane barrier. The permeability increases due to the increased solubility of the extract and the effect of penetration enhancers of the constituents of ME, in particular of Cremophor EL and Transcutol.

### 2.7. Transport Experiments with Caco-2 Cells

In order to complete the in vitro characterization of OE-ME, permeation studies were performed using a cell-based model. Caco-2 cells are considered the most predictive in vitro model to estimate not only passive intestinal diffusion, as previously described for PAMPA, but also active transport processes, paracellular permeability, and active efflux [66,67].

**Figure 6:**
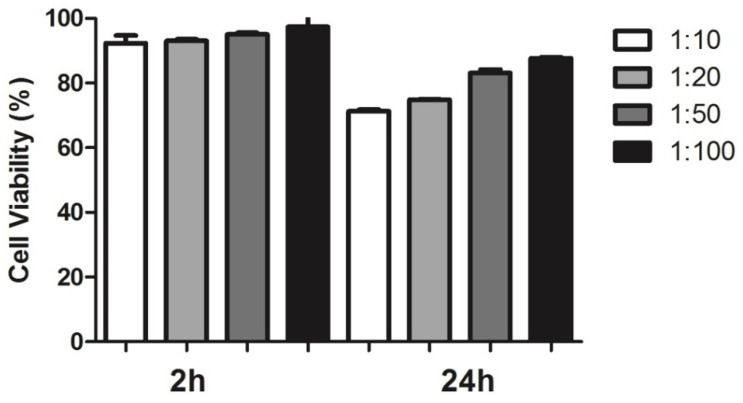
Viability of Caco-2 cells exposed to OE-ME (1:10 to 1:100), for 2 h.

To find the highest non/low toxic concentrations to be used in the transport experiments, the cytotoxicity of OE-ME was tested. For the MTS assay, the cells were incubated for 2 and 24 h at 37 °C with fresh medium containing OE-ME. The results are shown in Figure 6.

OE-ME loaded with 35 mg/mL of extract does not show any sign of cytotoxicity after 2 h of exposure, being the Caco-2 cellular viability close to 100% for all tested dilutions. After 24 h, the cell viability percentage was still good: for ME diluted 1:100, it was up to 90% compared to untreated cells, confirming that the formulation and the extract do not affect cell viability. The dilution 1:100 was selected for transport experiments, considering that a cell viability ˃80% is required for an acceptable in vitro assay [68]. In addition, the Lucifer yellow passage was less than 3%, indicating the integrity of the layer [69].

Caco-2 cells were exposed to OE-ME 1:100 or OE at the same concentration present in the ME formulation (35 mg/mL), for 2 h, in the AP chamber, while the BL was filled with culture medium.

The P_app_ of the OE solution was 16.14 ± 0.05 × 10^−6^ cm/s while the P_app_ of OE-ME was 26.99 ± 0.45 × 10^−6^ cm/s, *p* = 0.0571. By calculating the sum of phenolic compounds concentration in the incubation media of both chambers, we estimate a recovery up to 80%. The permeability coefficient of the ME was significantly higher than that of the unformulated OE.

Therefore, the formulation provided enhanced intestinal permeability of OE. This fact is probably due to the presence of surfactant used as a stabilizer of the internal phase. As previously reported, the surfactants enhance drug permeability in many ways, such as by increasing transcellular permeability and by inhibiting the efflux transport systems [70,71].

Moreover, non-ionic surfactants also contributed to increasing the contact time with the absorption site, and to increase endocytic and transcellular pathways by opening the tight junctions [72]. Many studies have been performed to elucidate the P-gp inhibition effect of Cremophor EL as well as other surfactants [73] and their mechanisms [74]. It was indicated that Cremophor EL and Transcutol may inhibit the function of P-gp by affecting membrane fluidity [40]. Cremophor could specifically bind to the hydrophobic domain of the P-gp that may change its secondary and/or tertiary structure and reduce its function [73]. Furthermore, the small dimensions of droplets of ME characterized by a big surface area increase solubility and absorption.

## 3. Materials and Methods

### 3.1. Chemicals

Ultrapure water was produced using the Milli-Q-system (Millipore SA, Molsheim, France). Acetonitrile of HPLC and HPLC-MS grades were from Panreac (Barcelona, Spain). Formic acid, hexane, and ethanol of analytical reagent grade were from Sigma-Aldrich (Steinheim, Germany). Standards of tyrosol (>99.5%, Sigma-Aldrich, Steinheim, Germany) and rutin (>99%), luteolin-7-*O*-glucoside (>98%), verbascoside (>99%), and oleuropein (>98%) from Extrasynthese (Genay, France) were used for quantitative analysis.

Cremophor EL, Tween 80, and Phosphate buffered saline BioPerformance Certified pH 7.4 (PBS), were obtained from Sigma Aldrich (Saint Louis, MO, USA) with the support of Sigma Aldrich Italia (Milan, Italy). Soluplus^®^ was a gift from BASF (Ludwigshafen, Germany) with the support of BASF Italia, BTC Chemical Distribution Unit (Cesano Maderno, Monza e Brianza, Italy). Captex 300, Captex 355, Labrasol ALF, Capryol 90, Transcutol HP, Labrafac, Labrafilm 1944, Labrafilm 2125, and Lauroglycol 90 were from Gattefossé (Saint Priest, France).

### 3.2. Olive Samples and Preparation of the Phenolic Extract

Samples of olive fruits (*Olea europaea* L.) from the Moraiolo cultivar were collected during the 2017 Summer before any stone lignification, particularly on the July 18th. Unripe olives were carry-on harvested from 10 selected plants in a farm located in Fiesole (Florence, Italy), which applied regular irrigation of orchards. About 1 kg of olives were picked along the whole circumference of the selected plants at a height of about 170 cm. As soon as they arrived in the laboratory, olives were deep-frozen in liquid N_2_ and immediately freeze-dried. The mean weight of olives was 0.493 g per olive, while the moisture content, evaluated as the water lost during the lyophilization process, was 57%. After lyophilization, the dried olives were minced in a laboratory miller (Zautec, Germany), thus obtaining about 430 g of a homogeneous olive powder.

Starting from this powdered material, a phenolic extract was prepared at laboratory scale as follow. Ten aliquots of 2.5 g of powder were weighted in plastic tubes and cold extracted twice with 40 mL of EtOH:H_2_O 80:20 *v*/*v*, homogenizing the mixture with ULTRA-TURRAX at 8117× *g* for 4 min. The obtained mixture was cold centrifuged for 10 min at 1167× *g*, and the recovered solution was defatted twice with 60 mL of hexane and then evaporated under vacuum. The dried extract was recovered with three aliquots of 16 mL, 8 mL, and 4 mL of water and with the aid of ultrasounds. The obtained solution was then freeze-dried, thus obtaining a dried phenolic extract. The yield of the extract was 40% on the initial powder base, and thus we obtained a total of approx. 10 g of the extract.

The extract was characterized by HPLC-DAD: 50 mg of dried extract were dissolved in 1 mL of MeOH:H_2_O 50:50 *v*/*v*, and the obtained solution was used for the chromatographic analysis.

### 3.3. HPLC-DAD Analysis

For the chromatographic analysis, an HP1100 liquid chromatograph coupled with DAD detector was used (Agilent Technologies, Palo Alto, CA, USA) as previously described [52]. A Poroshell 120, EC-C18 (150 mm × 3.0 mm id, 2.7 µm; Agilent Technologies, Palo Alto, CA, USA) column coupled with a pre-column of the same phase, working at 26 °C, was used for separation of phenolic compounds. Elution was performed at a flow rate of 0.4 mL/min, using (A) acetonitrile and (B) H_2_O (pH 3.2 by formic acid) as solvents for the mobile phase, with the following multistep linear gradient: solvent A was increased from 5% to 40% in 40 min, then remained at 40% for 5 min, increased to 100% in 5 min, and stayed at 100% for 3 min, then returned to 5% in 2 min. Total elution time of 55 min, equilibration time 10 min, injection volume, 2 μL. Chromatograms were recorded at 240, 280, and 330 nm.

Quantification of phenolic compounds was carried out using 5 six-points calibration lines, built using hydroalcoholic solutions of tyrosol (linearity range 0–1.21 µg, R^2^ = 0.9999), oleuropein (linearity range 0–3.16 µg, R^2^ = 0.9986), luteolin-7-*O*-glucoside (linearity range 0–1.57 µg, R^2^ = 0.9956), rutin (linearity range 0–1.25 µg, R^2^ = 0.9975) and verbascoside (linearity range 0–1.96 µg, R^2^ = 0.9996). Limits of quantification (LOQ) were evaluated according to a previous study [75], using the above standards, and all the obtained values were lower than 0.1 mg/kg. Tyrosol and hydroxytyrosol were expressed as mg_tyr_ kg^−1^, rutin as mg_rut_ kg^−1^, luteolin-7-*O*-glucoside as mg_lut_ kg^−1^, verbascoside as mg_ver_ kg^−1^, and oleuropein, comselogoside, and ligstroside as mg_ole_ kg^−1^. Finally, the total phenolic content was calculated as sum of the content of the previous phenolic compounds.

### 3.4. Solubility Study

The solubility of the powdered OE in different vehicles was evaluated by adding an excess of OE to 5 mL of each of the tested solvent/tenside: Water, Capryol 90, Captex 300, Captex 355, Labrafac, Labrafilm 1944, Labrafilm 2125, Labrasol ALF, Lauroglycol 90, Transcutol, and Cremophor EL. Each mixture of solvent and OE was stirred for 24 h at 25 °C, then it was centrifuged at 13,148× *g* for 10 min. After removing the precipitate, the supernatant was diluted with EtOH and analyzed by HPLC-DAD to determine the concentration of the phenolic compounds from the OE. The analyses were performed in triplicate.

### 3.5. Pseudo-Ternary Phase Diagram Construction

The Chemix School (version 3.60, Arne Standnes, Norway) was used for constructing the pseudo-ternary phase diagram following the water titration method, in order to define the area of existence of the ME [76]. The selected surfactant and co-surfactant were mixed at various ratios (S_mix_), for each of which the pseudo-ternary phase diagram was built using different weight ratios oil-phase/S_mix_: 0:100, 5:95, 10:90, 20:80, 30:70, 40:60, 50:50, 60:40, 70:30, 80:20, and 90:10. Each of the mixtures obtained using these different oil-phase/S_mix_ ratios were tested adding water dropwise to each blend, under vigorous stirring at room temperature. During water addition, the change in samples appearance was monitored to determine if transparent ME, emulsion, or gel was present.

### 3.6. Solubility of the Olive Extract into the Microemulsion

After the elaboration of a pseudo-ternary phase diagram, the maximum loading content of olive extract into the formulation was evaluated adding increasing amount of extract powder to the ME under stirring. The sample was gently stirred for 24 h at room temperature, protected from the light. Afterwards, excess extract was removed by centrifugation at 13,148× *g* for 10 min and the supernatant was analysed by HPLC after dilution with ethanol. The analyses were performed in triplicate.

### 3.7. Characterization of Microemulsion

#### 3.7.1. Particle Size and ζ-Potential Measurements

Droplet sizes of the developed ME were measured by a dynamic light scattering (DLS), Ζsizer Nano series ZS90 (Malvern Instruments, Malvern, UK) equipped with a JDS Uniphase 22 mW He-Ne laser operating at 632.8 nm, an optical fiber-based detector, a digital LV/LSE-5003 correlator, and a temperature controller (Julabo water-bath) set at 25 °C. Time correlation functions were analysed to obtain the hydrodynamic diameter of the particles (Zh) and the particle size distribution (polydispersity index, PdI) using the ALV-60 × 0 software V.3.X provided by Malvern. Autocorrelation functions were analysed by the Cumulants method, fitting a single exponential to the correlation function to obtain particle size distribution. Scattering was measured in an optical quality 4 mL borosilicate cell at a 90° angle, diluting the samples in distilled water. ζ-potential was measured using the same instrument; for all samples, an average of three measurements at stationary level was taken. The temperature was kept constant at 25 °C by a Haake temperature controller. ζ-potential was calculated from the electrophoretic mobility, using the Henry correction to Smoluchowski’s equation.

#### 3.7.2. In Vitro Release Study

The release studies of OE from ME in comparison to OE hydroalcoholic solution (EtOH:H_2_O 70:30) were carried out with the dialysis bag method (regenerated cellulose dialysis membranes, Spectrum Laboratories, Inc., Breda, The Netherlands, MWCO 12–14 kD). Two mL of the OE-ME or the solution were placed into dialysis membranes, sealed, and then immersed into 200 mL of the release medium at 37 °C under magnetic stirring. Simulated gastric fluid (SGF, pH 1.2), simulated intestinal fluid (SIF pH 6.8), and PBS were used as release media. The composition of the gastric fluid was 2 g of NaCl and 7 mL of HCl per liter of deionized water. The intestinal fluid was composed of 6.805 g of KH_2_PO_4_ and 0.896 g of NaOH per liter of deionized water [77].

At predetermined time intervals, 1 mL of each release medium was withdrawn and replaced with equal volume of fresh solution. The total phenolic concentration in samples was finally determined by HPLC. All studies were performed in triplicate.

### 3.8. Chemical and Physical Stability during Storage

To estimate the shelf life, the OE-ME was stored in sealed glass containers at 4 °C for one month. Chemical and physical stabilities were checked periodically by monitoring transparency, phase separation, colour variation as the changes in particle size, homogeneity, ζ-potential, and extract concentration by DLS and HPLC/DAD analyses.

### 3.9. In Vitro Parallel Artificial Membrane Permeability Assay (PAMPA)

The test was carried out in a 96-well, MultiScreen-IP PAMPA (Millipore corporation) filter plate. The ability of compounds to diffuse from a donor compartment into an acceptor compartment was evaluated, by placing a polyvinylidene difluoride (PVDF) membrane filter pretreated with a lipid-containing organic solvent between the two compartments. DMSO/PBS (0.05 mL/mL, pH 7.4) mixture was used as receptor buffer. The artificial membrane added to each filter consisted of a combination of lecithin and cholesterol, 10 g/L and 8 g/L, respectively, in 1,7-octadiene. Immediately after the deposition of the lipid solution (5 µL), 250 µL of extract solution and OE-ME were added to each well of the donor plate. Each receptor plate was filled with 250 µL of EtOH/PBS buffer. Afterwards, the donor plate was placed into the receptor plate, ensuring that the underside of the membrane was in contact with the buffer. The plate assembly was covered and incubated at room temperature for 2 h.

Then, the samples were withdrawn, diluted with ethanol, centrifuged for 10 min at 13,148× *g*, and the OE concentration was determined by HPLC. The *P_e_* (cm/s) was calculated according to the following equation:

(1) $$P_{e}=\frac{-ln\left[1-\frac{C_{At}}{C_{eq}}\right]}{A\left(\frac{1}{V_{D}}+\frac{1}{V_{A}}\right)t}$$

where A is the active surface area (0.3 cm^2^ × apparent porosity of the filter), *V_D_* and *V_A_* the well volume of the donor and acceptor plate (mL), respectively, t the incubation time (s), and *C_At_* and *C_Dt_* the concentration of APP in the acceptor and donor plate at time t, respectively. *C_eq_* was calculated according to:

(2) $$C_{eq}=\frac{\left[C_{Dt}\times V_{D}+C_{At}\times V_{A}\right]}{V_{A}+V_{D}}$$

The experiments were performed in quadruplicate.

### 3.10. MTS Assay for Cell Viability

Viability analyses were performed using Cell Titer 96 Aqueous One solution cell proliferation (3-(4,5-dimethylthiazol-2-yl)-5-(3-carboxymethoxyphenyl)-2-(4-sulfophenyl)-2*H*- tetrazolium) (MTS) assay kit (Promega Madison, WI, USA). Briefly, Caco-2 cells were transferred to flat bottom 96-well tissue culture plates (Corning, NY, USA) at a seeding density of 5 × 10^3^ cells/well and allowed to grow for 24 h under the conditions detailed above. For the MTS assay, the culture medium was removed and replaced with fresh medium containing OE-ME and the cells were incubated for 2 and 24 h. Then the cells were exposed to MTS solution and allowed incubating for 2 and 24 h at 37 °C. The product of the reaction was measured at 490 nm using a spectrophotometer (Multilabel Counter 1240 Victor 3, Perkin Elmer, Milan, Italy). The cell death was expressed as a percentage of values obtained from control, untreated cells, calculated from three replicates of each ME dilution.

### 3.11. Cell Culture for Transport Studies

For transport studies, cells were seeded at 50,000 cells/well in cell, culture inserts with polyethylene terephthalate (PET) membranes (BRAND, Italy). Culture medium (DMEM) was added to apical (AP) and basolateral (BL) side and was replaced every day for the first week and daily thereafter. Cells were left to differentiate for 18–21 days.

#### 3.11.1. Monolayer Integrity

The integrity of the layer was evaluated with the Lucifer Yellow (LY) permeability assay. LY was diluted in the transport buffer (Hank’s Balanced Salt Solution, HBSS, with Ca^2+^, Mg^2+^, 25m M HEPES, pH 7.4) and added to the AP compartment at a final concentration of 100 µM. After incubation at 37 °C for 1h, the HBSS in the BL chamber was collected, and the concentration of LY determined by using 485 nm excitation and 530 nm emission on a fluorescence plate reader (Multilabel Counter 1240 Victor 3, Perkin Elmer). The percentage of AP to BL permeability was calculated, according to the following equation:

% permeability = (Fluorescence in the BL-blank)/(Fluorescence LY-blank) ∗ 100(3)

The critical maximum flux of LY to identify leaky monolayers was fixed to be less than 3% of starting concentration.

#### 3.11.2. Transport Experiments

Transport studies were performed according to Hubatsch et al., (2007) [64]. Briefly, the culture medium (DMEM) was replaced with preheated (37 °C) transport HBSS medium supplemented with 25 mM HEPES (pH 7.4). After that, the cell monolayer was equilibrated for 30 min at 37 °C. Caco-2 cells were then exposed for 2 h with OE-ME 1:100 or OE not formulated, in the apical (AP) chamber, while the basolateral (BL) chamber contained only HBSS.

At predetermined time intervals, 0.3 mL of medium in the BL side was taken for HPLC analyses and replaced with the same volume of fresh HBSS. At the end of the experiment, the integrity of the layer was re-evaluated with the LY permeability assay as described above.

The apparent permeability coefficient (*P_app_*, cm/s) was calculated according to the following equation:

(4) $$P_{app}=V_{D}∕\left(A\times M_{D}\right)\times \left(∆M_{R}/∆t\right)$$

where: *V_D_* = apical (donor) volume (mL), *M_D_* = apical (donor) amount, and Δ*M_R_*/Δ*t* = change in amount of compound in receiver compartment over time.

### 3.12. Statistical Analysis

The experiments were performed at least in triplicate. The results of the quantitative analysis of OE, the physicochemical characterization of ME and the in vitro tests were expressed as means ± SD and analyzed by Mann–Whitney test. All analyses were carried out using GraphPad Prism 7.0 (GraphPad Software, San Diego, CA, USA). A *p* value of 0.05 was considered significant.

## 4. Conclusions

A microemulsion intended for preparation of oral dosage form and suitable for delivering an OE was developed for the first time. The olive extract was obtained in the powder form from Tuscan olives of the Moraiolo cultivar harvested before any stone lignification and immediately lyophilized after freezing them with liquid nitrogen. It is characterized by a unique phytocomplex extremely rich in the phenolic compounds ligstroside, verbascoside, and particularly oleuropein, making it different from the commercial extracts obtained from olive leaves during the pruning period. Finally, the use of a well-characterized extract as a whole is economically advantageous and often more effective than the use of a single isolated component. A microemulsion is a formulation well suited to the delivery of not only single compounds, but also a phytocomplex. The microemulsion developed is suitable in terms of physico-chemical characteristics for oral administration and shows appropriate chemical and physical stability for over 30 days during storage at 4 °C. ME has a good solubilizing effect on the olive extract, with a pronounced influence on its permeability, with respect to the unformulated extract. Caco-2 test confirmed PAMPA results and these in vitro assays revealed that the formulation increases the in vitro passive and active permeation of extract.

## Figures and Tables

**Table 1:** Phenolic composition of the olive extract. Data are expressed as the mean ± SD from three independent determinations.

Compound	Extract (mg/kg)
hydroxytyrosol (mg/kg)	5424.7 ± 270.8
tyrosol (mg/kg)	655.2 ± 46.4
rutin (mg/kg)	6535.5 ± 286.6
luteolin-7-*O*-glucoside (mg/kg)	1696.2 ± 613.6
verbascoside (mg/kg)	23786.9 ± 628.7
oleuropein (mg/kg)	310187.5 ± 2203.1
comselogoside (mg/kg)	7399.8 ± 738.2
ligstroside (mg/kg)	31477.1 ± 1375.8
Total phenolic compounds (mg/kg)	387162.7 ± 4362.8

**Table 2:** Solubility of the main phenolic compounds of the olive extract in different vehicles. Data are expressed as mean ± sd from three independent measurements.

	Verbascoside	Oleuropein	Ligstroside	Total Phenolic Content
	(mg/mL)	(mg/mL)	(mg/mL)	(mg/mL)
Water	0.243 ± 0.007	3.081 ± 0.007	0.314 ± 0.003	3.816 ± 0.071
Capryol 90	0.060 ± 0.009	1.079 ± 0.074	0.115 ± 0.001	1.292 ± 0.107
Captex 300	nd	0.058 ± 0.012	0.014 ± 0.001	0.074 ± 0.013
Captex 355	nd	0.038 ± 0.005	0.012 ± 0.001	0.052 ± 0.006
Labrafac	nd	0.032 ± 0.017	0.007 ± 0.003	0.039 ± 0.020
Labrafilm 1944	0.010 ± 0.001	0.411 ± 0.007	0.050 ± 0.003	0.483 ± 0.002
Labrafilm 2125	0.010 ± 0.001	0.456 ± 0.007	0.070 ± 0.022	0.545 ± 0.033
Labrasol ALF	0.411 ± 0.007	5.331 ± 0.010	0.526 ± 0.011	6.582 ± 0.057
Lauroglycol 90	0.018 ± 0.004	0.611 ± 0.001	0.069 ± 0.002	0.729 ± 0.009
Transcutol	0.702 ± 0.007	9.036 ± 0.005	0.896 ± 0.025	11.081 ± 0.046
Cremophor EL	0.326 ± 0.040	5.269 ± 0.312	0.510 ± 0.047	6.320 ± 0.419

nd: not defined.

**Table 3:** Physical characterization of ME and olive extract-ME. Results are expressed as means ± standard deviation of at least three experiments.

Sample	Size (nm)	PdI	ζ-Potential (mV)
ME	13.15 ± 0.19	0.14 ± 0.01	−1.23 ± 0.17
OE-ME	14.03 ± 1.36	0.20 ± 0.08	−1.16 ± 0.48
